# Religious/spiritual beliefs and behaviours and study participation in a prospective cohort study (ALSPAC) in Southwest England

**DOI:** 10.12688/wellcomeopenres.17975.1

**Published:** 2022-07-12

**Authors:** Jimmy Morgan, Isaac Halstead, Kate Northstone, Daniel Major-Smith

**Affiliations:** 1 Centre for Academic Child Health, Population Health Sciences, University of Bristol, Bristol, UK; 2 Population Health Sciences, University of Bristol, Bristol, UK; 3 MRC Integrative Epidemiology Unit, University of Bristol, Bristol, UK

**Keywords:** ALSPAC, religion, selection bias, study participation, longitudinal study

## Abstract

*Background*

Longitudinal studies are key to understanding risk factors for health, well-being, and disease, yet associations may be biased if study invitation and participation are non-random. Religious/spiritual beliefs and behaviours (RSBB) are increasingly recognised as having potentially important relationships with health. However, it is unclear whether RSBB is associated with study participation. We examine whether RSBB is associated with participation in the longitudinal birth cohort ALSPAC (Avon Longitudinal Study of Parents and Children).

*Methods*

Three RSBB factors were used: religious belief (belief in God/a divine power; yes/not sure/no), religious affiliation (Christian/none/other), and religious attendance (frequency of attendance at a place of worship). Participation was measured in three ways: i) total number of questionnaires/clinics completed; ii) completion of the most recent questionnaire (in 2020); and iii) length of participation. Analyses were repeated for the ALSPAC mothers, their partners, and the study children, and were adjusted for relevant socio-demographic confounders.

*Results*

Religious attendance was positively associated with participation in all adjusted models in all three cohorts. For example, study mothers who attended a place of worship at least once a month on average completed two more questionnaires (out of a possible 50), had 50% greater odds of having completed the most recent questionnaire, and had 25% reduced risk of drop-out, relative to those who did not attend a place of worship. In the adjusted analyses, religious belief and attendance were not associated with participation. However, the majority of unadjusted models showed associations between RSBB and participation.

*Conclusion*

After adjusting for confounders, religious attendance – not religious belief or affiliation – was associated with participation in ALSPAC. These results indicate that use of RSBB variables (and religious attendance in particular) may result in selection bias and spurious associations; these potential biases should be explored and discussed in future studies using these data.

## Introduction

Longitudinal studies are critical to understanding risk factors for health, well-being, and disease. However, longitudinal studies frequently suffer from loss of participation over time, and this drop-out is likely to be non-random, based on characteristics such as age, sex, and socioeconomic background, among others. If drop-out is related to the exposure and the outcome (or just the outcome ( Hernán, 2017)) in a given analysis, this may result in selection bias (a form of collider bias), potentially resulting in biased associations between the exposure and outcome ( Griffith *et al.*, 2020; Hernán & Robins, 2020; Hughes *et al.*, 2019; Munafò *et al.*, 2018).

In the present study (ALSPAC; the Avon Longitudinal Study of Parents and Children), the original pregnancy cohort was broadly representative of the population of pregnant women in 1990–1992 around Bristol (UK) ( Fraser *et al.*, 2013), but continued participation in the study after 30 years is likely to be non-random. Selection bias has been explored in ALSPAC previously, with factors such as lower socioeconomic position, other than white ethnicity and younger age at birth associated with lower rates of continued participation ( Boyd *et al.,* 2013; Fraser *et al*., 2013). Further research has explored potential selection biases in ALSPAC in more detail, including in relation to COVID-19 data collection ( Fernández-Sanlés *et al.*, 2021), various polygenic risk scores associated with participation ( Taylor *et al.*, 2018), and using linked education and primary care data to overcome potential selection bias ( Cornish *et al.*, 2021).

In this paper, we aim to explore whether religious/spiritual beliefs and behaviours (RSBB) are associated with continued participation in ALSPAC. In epidemiology, religion is often an overlooked factor but its relationship with health has been increasingly recognized in recent years ( Koenig *et al.*, 2012; Shields & Balboni, 2020; VanderWeele, 2017). Additionally, RSBB is central to a project understanding the associations between religion and health and will inform future research in this project, as well as other research using ALSPAC’s RSBB data ( Iles-Caven *et al.*, 2021b; Iles-Caven *et al.*, 2021a). This will also provide information for other longitudinal population-based studies as to whether RSBB may be associated with participation and potential selection effects.

It is not known whether religiosity influences continued participation in longitudinal studies as, to the best of our knowledge, no studies have investigated this question. The direction of this effect (if any) could plausibly be in either direction. For instance, as religion and science have some conflicting world views, and with religion influencing an individual’s acceptance of and attitudes towards science ( Jost *et al.*, 2009; O’Brien & Noy, 2015), this conflict may potentially impact attitudes towards research and participation in scientific studies ( Ecklund *et al.*, 2016; McPhetres & Zuckerman, 2018). However, this likely depends on each individual’s perception of the study; if participants view the study as more of a public endeavour or a study of health, rather than a strictly scientific enterprise, then religiosity may not have a negative impact on participation.

If continued participation is felt to be a public endeavour, this may have the opposite effect on participation, as religious individuals often exhibit more prosocial behaviours than non-religious people ( Hardy & Carlo, 2005; Oviedo, 2016; Schulz *et al.*, 2019). Current literature suggests that religion may facilitate cooperation (helping others at a cost to self in terms of time and energy), through mechanisms such as prosocial religious norms, supernatural punishment and engagement with cooperative religious networks ( Henrich, 2021; Lewis *et al.*, 2013; Norenzayan *et al.*, 2016; Purzycki *et al.*, 2016). According to this hypothesis, taking part in longitudinal health studies could be seen as a form of cooperation, meaning that those who are more religious may be more likely to continue participating. The Office of National Statistics (ONS) 2020 study of religion and political and social participation showed that in the UK there are substantial differences in prosocial behaviours (such as volunteering) between different religious affiliations ( Office for National Statistics, 2020).

We aim to explore this topic using ALSPAC, a large prospective birth cohort study centered in Bristol, UK. Our main research question is: Does religiosity affect study participation? This will add to the understanding of factors affecting participation in longitudinal population studies and influence the interpretation of subsequent studies exploring RSBB and health outcomes. As there is little research into the potential association between religiosity and study participation, we aim to explore this using a geographically representative longitudinal population-based study with a large sample size ( *n*=~14,000). Specifically, we will explore whether various measures of RSBB (religious belief, religious affiliation, and religious attendance) are associated with study participation.

## Methods

### Participants

Pregnant women resident in Bristol and surrounding areas in the UK with expected delivery dates between 1 ^st^ April 1991 and 31 ^st^ December 1992 were invited to take part in the study. The initial number of pregnancies enrolled was 14,541, of which there were a total of 14,676 fetuses, resulting in 14,062 live births and 13,988 children who were alive at 1 year of age ( Boyd *et al.*, 2013; Fraser *et al.*, 2013; Northstone *et al.*, 2019). After removing pregnancies that did not result in a live birth (most being early miscarriages), removing one pregnancy if the mother had two pregnancies enrolled in ALSPAC, excluding mothers known to have died since the study began, and dropping observations for participants who had withdrawn consent for their data to be used, there were a total of 13,300 G0 (Generation-0) mothers and their associated partners. A possible 13,934 G1 (Generation-1) offspring were included, consisting of all children alive at 1 year of age who had not withdrawn consent. Partners/fathers (henceforth partners) were not formally enrolled into ALSPAC but were given partner-based questionnaires by the mother (if she had a partner and chose to share the questionnaire). This means that partner-based questionnaires may not have been completed by the same partner over time (although numbers of such cases are likely to be relatively small); for the purposes of this study, we assume that the identity of the partner is the same over all waves of data collection used. Only participants with valid RSBB data were included in these analyses. For instance, of the potential 13,300 G0 mothers, 11,758 had data regarding religious beliefs (See Table 1 for full sample sizes).

**Table 1.** Descriptive statistics for the religious and spiritual beliefs and behaviour variables for G0 mothers and partners.

		Mothers, n (%)	Partners, n (%)
**Belief in God/divine power**	*Yes*	5,858 (49.82%)	3,422 (36.81%)
	*Not sure*	4,159 (35.37%)	3,216 (34.60%)
	*No*	1,741 (14.81%)	2,658 (28.59%)
	*Total*	11,758	9,296
Missing Data		1,542 (11.59%)	4,004 (30.11%)
**Religious affiliation**	*None*	1,779 (15.32%)	2,367 (25.86%)
	*Christian*	9,347 (80.47%)	6,305 (68.89%)
	*Other*	490 (4.22%)	480 (5.24%)
	*Total*	11,616	9,152
Missing Data		1,684 (12.66%)	4,148 (31.19%)
**Attendance at church/place of worship**	*Min once a month*	1,642 (14.29%)	938 (10.30%)
	*Min once a year*	3,357 (29.21%)	2,380 (26.14%)
	*Not at all*	6,494 (56.50%)	5,786 (63.55%)
	*Total*	11,493	9,104
Missing Data		1,807 (13.59%)	4,196 (31.55%)

Ethical approval for the study was obtained from the ALSPAC Ethics and Law Committee (ALEC) and the Local Research Ethics Committee. Informed consent for the use of data collected *via* questionnaires and clinics was obtained from participants following the recommendations of the ALSPAC Ethics and Law Committee at the time. Please note that the study website contains details of all the data that is available through a fully searchable data dictionary and variable search tool: http://www.bristol.ac.uk/alspac/researchers/our-data/. From 2014 onwards, study data were collected and managed using REDCap electronic data capture tools hosted at the University of Bristol ( Harris *et al.*, 2009). REDCap (Research Electronic Data Capture) is a secure, web-based software platform designed to support data capture for research studies.

### Exposure variables: RSBB

A questionnaire completed during pregnancy (mean mother’s age at birth was 27.9 [SD=4.95]; mean partner’s age in pregnancy was 30.3 [SD=5.72]) asked three questions assessing RSBB: “Do you believe in God/a divine power?” (coded as yes/no/not sure), “What sort of religious faith would you say you had?” (coded as Christian/none/other), and “Do you go to a place of worship?” (coded as more than once a month/more than once a year/not at all). We refer to these as ‘religious belief’, ‘religious affiliation’, and ‘religious attendance’, respectively. The G0 mothers’ RSBB data was used as a proxy for G1 religiosity, as comparable RSBB questions for G1 participants were not asked until adulthood, meaning it would not be possible to assess whether RSBB was associated with continued G1 participation using these data. These variables were chosen to cover a range of religious beliefs and behaviours.

### Outcome measures: ALSPAC participation

Table S1 in the extended data ( Morgan *et al*., 2022) lists the questionnaires and clinics used in the study, along with the variables that indicate completion. The outcome measures used in this study were derived in the same way as Taylor *et al*. ( Taylor *et al.*, 2018). First, we summed the number of completed questionnaires and clinics attended by each participant in each cohort. Mothers were asked to complete questionnaires about herself and her child in the early years of the study, both are included in the G0 mother’s outcome. Only completion of clinics and questionnaires that all participants were invited to were used ( *i.e*., substudies which focussed on a small group of participants were not included). A total of 50 questionnaires and clinics could have been completed/attended for G0 Mothers, 19 for G0 partners, and 43 for G1 children.

In addition to this continuous variable, a binary outcome measure of most recent questionnaire completion was also used (questionnaire Y for G0 mothers, questionnaire FC for G0 partners, and questionnaire YPH for G1 children; all completed in 2020). We also conducted a survival analysis (detailed below) with the length of study participation as the outcome variable. This was defined as the period in months from when the study child was born to the most recent questionnaire or clinic completed by each of the three cohorts. Note that this ‘length of study participation’ variable does not take study engagement prior to most recent questionnaire completion into account; that is, a participant who participated in all data collections would be coded identically to a participant who only participated in the first and last data collections (the continuous ‘total number of data collections completed’ variable would be able to differentiate these two cases, however).

### Confounder variables

We selected the following confounder variables as potentially causing both the exposure (RSBB) and the outcome (study participation), therefore producing biased associations if not adjusted for. These confounders were selected based on our subject knowledge, existing literature, and logical considerations. For mothers, we adjusted for: age at birth of the study child, housing status, marital status, parity, financial difficulties, highest education qualification, ethnic group, urban/rural location, and Index of Multiple Deprivation (IMD). For partners we adjusted for: age in pregnancy, ethnic group, G0 mother’s financial difficulties score, marital status, highest educational achievement, the G0 mother’s urban/rural location, and the G0 mother’s IMD (on the assumption that G0 mothers and partners lived together, as these variables were not available for partners). All variables used in G0 analysis were collected during pregnancy. The covariates adjusted for in G1 analyses were: mother’s age at birth, mother’s highest educational qualification, sex, mother’s IMD, mother’s urban/rural location, parental financial difficulties, mother’s housing status, mother’s marital status, mother’s parity, and child ethnicity. Variable coding and descriptive statistics for these variables can be found in the supplementary information (tables S2–S4 in the extended data ( Morgan *et al*., 2022)).

### Analysis

All analyses were conducted in Stata v17 (RRID:SCR_012763) and were performed on each of the cohorts (G0 mothers, G0 partners and G1 children) separately using the outcomes, exposures and confounders detailed above. As there were three RSBB exposures (religious belief, religious affiliation, and religious attendance), each of the analyses below were repeated for each exposure. Analyses were conducted in both unadjusted models and fully adjusted models ( *i.e*., multivariable models with the exposure and all potential confounders included as covariates).

The first analysis was a linear regression, with the total number of completed questionnaires/clinics as the outcome. As this continuous ‘participation’ variable was found to have an approximately uniform distribution for all cohorts, violating an assumption of linear regressions, we also categorised this outcome into groups as a sensitivity analysis. The sensitivity analysis was an ordinal regression with the continuous outcome being split into groups of number of questionnaires/clinics. These groups were 0–10, 11–20, 21–30, 31–40, 41–49, and 50 for G0 mothers; 1–4, 5–9, 10–14, and 15–19 for G0 partners; and 0, 1–10, 11–20, 21–30, and 31–43 for G1. The proportional odds assumption of ordinal models was explored using a Brant test.

Second, logistic regressions were performed with completion of the most recent questionnaire (table S1) as the outcome. For ordinal and logistic models, predicted probabilities for each outcome category were calculated alongside the odds ratios for a more intuitive interpretation of effect sizes.

Finally, a survival analysis (Cox regression) was performed with survival time being the length of time between the G1 child’s birth and the last completed questionnaire (in months; if mothers or partners only completed pregnancy questionnaires, they were coded as ‘0.01’, to avoid zeros and negative values). Participants who did not complete any questionnaires or did not have any ages at completion (due to missing data) were excluded from the survival analyses (15 G0 mothers, 2,185 G0 partners and 2,417 G1 children). Participants who were still active in ALSPAC were censored based on when they completed the most recent questionnaire. A Kaplan–Meier survival curve was plotted for the outcomes and exposures for each cohort along with Nelson–Aalen cumulative hazard plots and Schoenfeld residuals’ tests to assess the proportional hazards assumption.

Two further sensitivity analyses were conducted. First, if the Brant test for the ordinal logistic regression indicated that the proportional odds assumption was violated (using a threshold of *p*<0.05), we used a generalized ordinal logistic regression, which relaxes this assumption, to examine whether these results were robust (using the Stata package ‘gologit2’) ( Williams, 2006). The second sensitivity analysis was for the survival analysis: rather than defining ‘active participation’ for censoring based on just completing the most recent data collection event, we also defined censoring based on completion of either of the two or three most recent data collections.

While all these analyses are similar, they each answer a slightly different question, which together will provide a more complete picture of continued ALSPAC participation. For instance, the linear/ordinal models examine overall levels of participation throughout the study, the logistic models assess recent participation, and the survival analysis explores length of participation. In addition to providing a broader assessment of participation, if all analyses report comparable results, this gives more confidence that the results are robust to different model specifications.

## Results

### Exposure characteristics

Descriptive statistics for the RSBB exposures are shown in Table 1. Differences between the sexes are apparent, with 50% of mothers, compared to 37% of partners believing in God/a divine power. This difference continued with the other exposures, with 80% of mothers identifying as Christian, 15% considering themselves non-religious and 4% as another religion, compared to 69% of partners identifying as Christian, 26% as non-religious, and 5% as other. The ‘other’ religion group consisted of those identifying themselves as “other” as well as Jewish, Buddhist, Sikh, Hindu, Muslim, and Rastafarian (these were grouped together due to small sample sizes). These differences continue with place of worship attendance, as 56% of mothers compared to 64% of partners state they never attend a place of worship.

### Outcome characteristics

**Figure 1.**
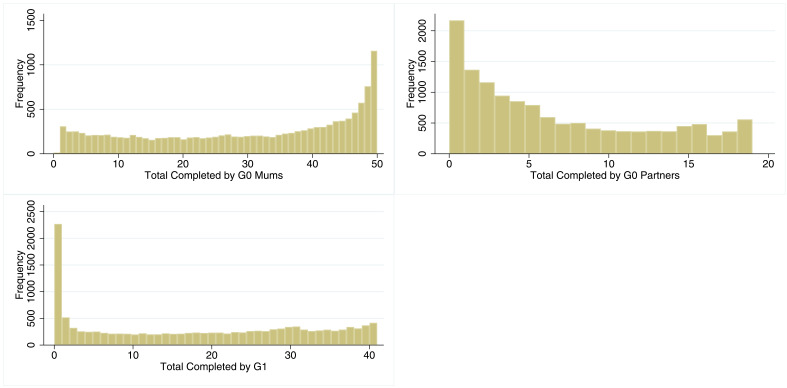
Histograms of total number of studies completed in all three cohorts. n for G0 = 13,300. n for G1 = 13,934.

The overall levels of participation for each cohort are shown in Figure 1. The histogram for G0 mothers shows a predominantly uniform distribution, with a large number of participants completing most of the questionnaires and clinics (median=34; IQR=16–46). The distribution of study participation for G0 partners is also somewhat uniform but skewed towards lower values (median=5; IQR=1–11). The distribution of G1 participation takes on a similar form with a uniform distribution but with a spike at ‘0’ (17% did not complete a single questionnaire; median=19; IQR=3–32). Grouped categories of participation are displayed in Table 2. 34% of G0 mothers, 15% of G0 partners, and 30% of G1 children completed most recent questionnaire.

**Table 2.** The number of participants in each group of ‘overall study participation’ for each of the cohorts.

Number of Questionnaires/Clinics completed by G0 Mothers	Frequency (n)	Percentage
0 – 10	2,281	17.15%
11 – 20	1,807	13.59%
21 – 30	1,929	14.50%
31 – 40	2,269	17.06%
41 – 49	3,856	28.99%
50	1,158	8.71%
Total	13,300	-
Number of Questionnaires/Clinics completed by G0 Partners	Frequency (n)	Percentage
0 – 4	6,501	48.88%
5 – 9	2,788	20.96%
10 – 14	1,851	13.92%
15 – 19	2,160	16.24%
Total	13,300	-
Number of Questionnaires/Clinics completed by G1	Frequency	Percentage
0	2,403	17.25%
1 – 10	2,820	20.24%
11 – 20	2,304	16.54%
21 – 30	2,814	20.20%
31 – 41	3,593	25.79%
Total	13,934	-

### Linear regression

**Figure 2.**
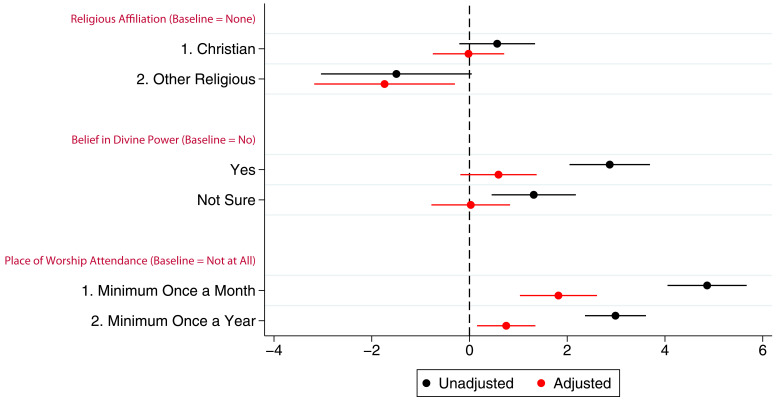
Coefficient plot of the three RSBB exposures with G0 mother study participation as the outcome in adjusted and unadjusted linear regression models. The x-axis shows the number of additional questionnaires/clinics completed on average compared to baseline (n for religious affiliation = 9,252, n for belief in a divine power = 9,358, n for place of worship attendance = 9,166). Error bars denote 95% confidence Intervals.

Figure 2 presents the coefficients (with 95% confidence intervals) from linear models for the associations between G0 mothers’ RSBB and study participation. In this cohort, before adjustment, both believing in and not being sure of a divine power had a positive association with study participation. However, once potential confounders were adjusted for, the association was no longer evident. Compared to non-religious participants, identifying as Christian had no association with study participation in either unadjusted or adjusted analyses, although ‘other’ religious affiliations were slightly negatively associated with participation; G0 mothers identifying as an ‘other’ religion completed one fewer questionnaire/clinic on average. Frequency of religious attendance was strongly associated with number of questionnaires/clinics completed by G0 mothers; those attending a place of worship at least once a month completed two more questionnaires/clinics on average than individuals who never attended, after adjustment. Complete model outputs can be found in table S5.

**Figure 3.**
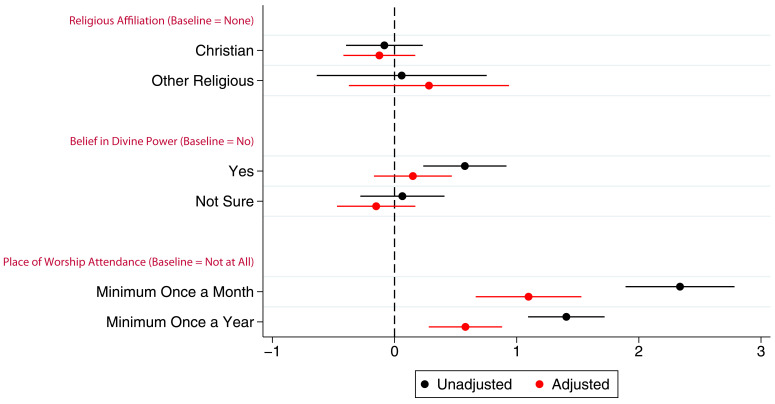
Coefficient plot of the three RSBB exposures with G0 partner study participation as the outcome in adjusted and unadjusted linear regression models. The x-axis shows the number of additional questionnaires/clinics completed on average compared to baseline (n for religious affiliation = 5,968, n for belief in a divine power = 6,064, n for place of worship attendance = 5,955). Error bars denote 95% confidence Intervals.

Associations between G0 partners’ study participation and RSBB exposure are shown in Figure 3 (full results in table S6). Similar to G0 mothers, there was little to no association between belief in a divine power and study participation particularly in the adjusted analyses, a pattern also observed with religious affiliation. In the adjusted analyses, G0 partner attendance at a place of worship at least once a month or once a year was associated with completing approximately one additional questionnaire/clinic on average.

**Figure 4.**
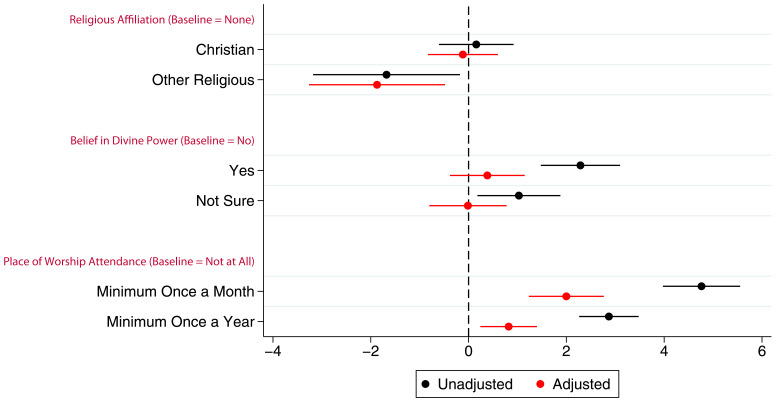
Coefficient plot of the three RSBB exposures with G1 study participation as the outcome in adjusted and unadjusted linear regression models. The x-axis shows the number of additional questionnaires/clinics completed on average compared to baseline (n for religious affiliation = 9,424, n for belief in a divine power = 9,530, n for place of worship attendance = 9,343). Error bars denote 95% confidence Intervals.

Associations between the G1 children’s study participation and maternal RSBB exposures are shown in Figure 4 (full results in table S7 in the extended data ( Morgan *et al.*, 2022)). The mothers’ identifying as Christian has little to no association with study participation of their G1 children. However, even after adjustment, G1 children whose mothers were categorised as ‘other’ religion completed around one fewer questionnaire/clinic on average. After adjustment, maternal belief in a divine power was not associated with study participation. However, G1 participants whose mothers attended a place of worship a minimum of once a month were associated with completing two more questionnaires on average compared to baseline after adjustment, whereas those whose parents who attended a minimum of once a year completed one more questionnaire on average. 

### Ordinal logistic regression

Results of the ordinal logistic regression for G0 mothers are presented in tables S8 (for coefficients) and S9 (predicted probabilities) in the extended data ( Morgan *et al.*, 2022), and are similar to the linear regression models, with little association evident between religious affiliation or belief in divine power and participation. However, there were strong positive associations with increased frequency attending a place of worship. As Brant tests indicated that the parallel regression assumption was violated (table S10 in the extended data ( Morgan *et al.*, 2022)) we also repeated these analyses using generalized ordinal models, finding comparable patterns of results (table S8 in the extended data ( Morgan *et al.*, 2022)). Results from G0 partners (tables S11, S12 and S10 in the extended data ( Morgan *et al.*, 2022) for coefficients, predicted probabilities and Brant test results, respectively) and G1 children (tables S13, S14, S10 in the extended data ( Morgan *et al.*, 2022) for coefficients, predicted probabilities and Brant test results, respectively) were similar to both the G0 mother results and those from the linear models, with strong associations only seen with religious affiliation and increased participation.

### Logistic regression

**Figure 5.**
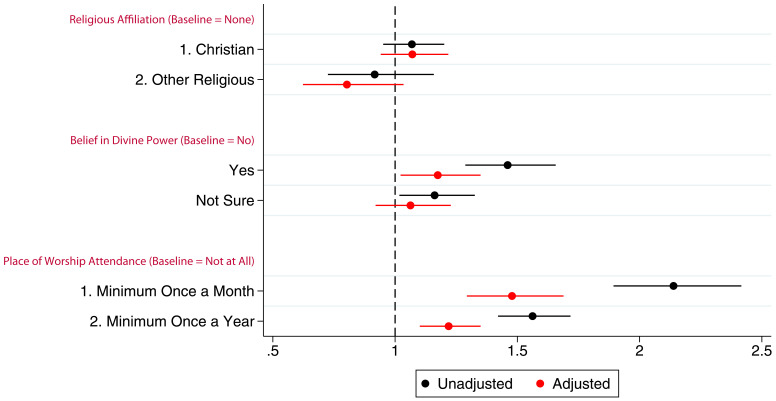
Plot of the odds ratios from a logistic regression of completing the most recent questionnaire by G0 mothers in ALSPAC with different RSBB exposures (n for religious affiliation = 9,525, n for belief in a divine power = 9,358, n for place of worship attendance = 9,166). Error bars denote 95% confidence Intervals.

As reported above, there were few strong associations between belief in a divine power or religious affiliation and completing the most recent questionnaire after adjustment in G0 mothers when adjusting for confounders ( Figure 5; full results in table S15 in the extended data ( Morgan *et al.*, 2022)). Attendance at a place of worship was again strongly associated with this outcome; in the adjusted model, attending at least once a month was associated with a 50% increase in the odds of completing the questionnaire, relative to those who did not attend. Based on the adjusted model, the predicted probabilities of completing said questionnaire were 46.5%, 42.2%, and 37.9% for attending at least once a month, at least once a year and never, respectively (see table S16 in the extended data ( Morgan *et al.*, 2022)). Similar results were found for G0 partners and G1 children (tables S17–S20 and figures S1 and S2 in the extended data ( Morgan *et al.*, 2022)).

### Survival analysis

**Figure 6.**
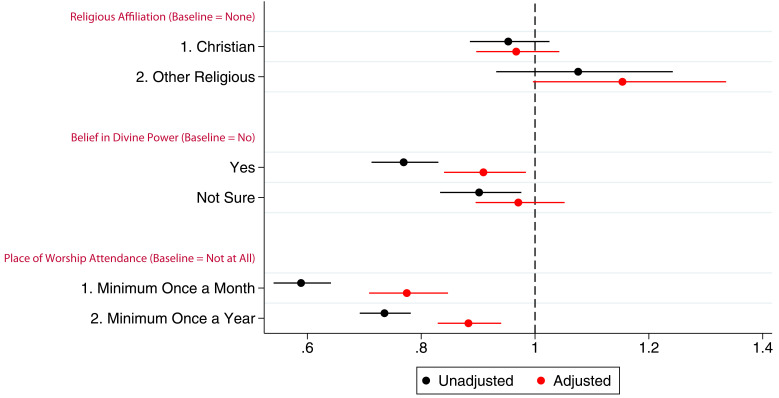
A hazard ratio plot of the three RSBB exposures and G0 mother participation from a cox regression. X-axis shows the hazard ratio for each model, values below 1 indicate a lower ‘hazard’ of leaving the study over time compared to baseline. (n for religious affiliation = 9,252, n for belief in a divine power = 9,538, n for place of worship attendance = 9,166). Error bars denote 95% confidence Intervals.

**Figure 7.**
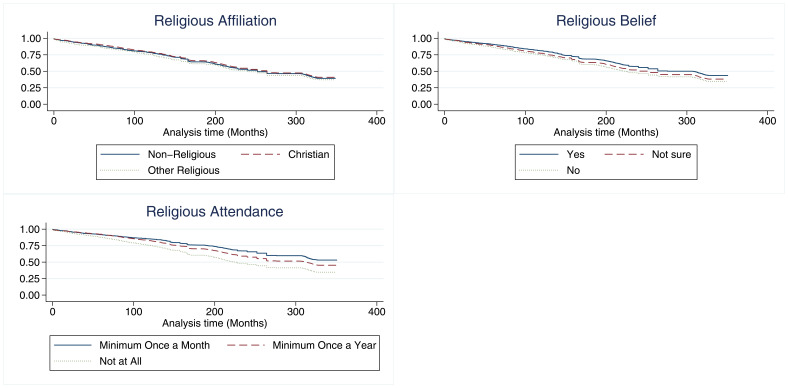
Kaplan-Meier survival estimates stratified by each RSBB exposure and G0 mothers’ completion of questionnaires/clinics over the course of ALSPAC. The X-axis shows the total time in months from the study child’s birth until the most recent questionnaire. The Y-axis shows the survival function for each stratum at a given timepoint. n for religious affiliation = 9,252, n for belief in a divine power = 9,538, n for place of worship attendance = 9,166.

The hazard ratios for the three RSBB exposures for G0 mother participation are shown in Figure 6. After adjusting for confounders, identifying as Christian was not associated with study drop-out. However, those who identified as an ‘other’ religion had a hazard ratio of 1.2, suggesting that these participants may be more likely to drop-out compared to the baseline of those with no religious affiliation. In adjusted analyses, belief in a divine power was weakly associated with continued study participation. Attending a place of worship a minimum of once a month was associated with a reduced hazard by around 0.75 times, relative to the baseline of never attending, whilst attending church at least once a year was also associated with a reduction in the hazard by around 0.9 times. Participants who attended a place of worship more regularly were therefore less likely to drop-out of the study. Figure 7 shows the unadjusted Kaplan–Meier survival estimate of this model (with Nelson–Aalen cumulative hazard plots in figure S3 in the extended data ( Morgan *et al.*, 2022)), full adjusted and unadjusted cox regression results can be found for all cohorts in the supplementary information (table S22 in the extended data ( Morgan *et al.*, 2022))

Religious affiliation and belief in a divine power were not associated with continued study participation for G0 partners or G1 children, after adjusting for appropriate confounders (tables S23 and S24, and figures S4 and S7 in the extended data ( Morgan *et al.*, 2022)). However, place of worship attendance was associated with length of participation, such that those attending more than once a month had a hazard ratio of around 0.75 times that of baseline for G0 partners and 0.80 for G1 children. Kaplan–Meier curves and Nelson–Aalen plots for these cohorts can be found in the supplementary information (figures S5, S6, S8 and S9 in the extended data ( Morgan *et al.*, 2022)).

In each cohort there are violations of the proportional hazards assumption, particularly for religious attendance (table S21 in the extended data ( Morgan *et al.*, 2022)). However, visual inspection of the cumulative hazard plots (figure S6 and S9 in the extended data ( Morgan *et al.*, 2022)) suggests that this assumption is not violated too severely and is unlikely to greatly impact the results reported here. Results from the two sets of sensitivity analyses with different right censoring conditions (figures S10–S15 in the extended data ( Morgan *et al.*, 2022)) were almost identical to the original survival analyses using just the most recent questionnaire to define ‘active participation’.

## Discussion

In a UK-based multigenerational birth cohort we have demonstrated that RSBB factors—and religious attendance in particular—are associated with i) overall study participation, ii) length of study participation, and iii) participation in recent questionnaires. Associations between participant RSBB characteristics and study participation are important as they can bias associations of any study using ALSPAC RSBB data as an exposure or outcome. A description of the relationships between study participation and RSBB data allows informed decisions to be made in future studies.

We found that participation in ALSPAC was positively associated with place of worship attendance after adjusting for various social, economic, and educational attainment confounding factors. Attending a place of worship a minimum of once a month had the greatest association with increased participation across all models, with attendance a minimum of once a year also being associated with an increase in study participation. In adjusted analyses, belief in a divine power and not being sure of a divine power showed little to no association with study participation compared to the baseline of no belief. Similarly, identifying as Christian had seemingly no association with ALSPAC participation compared to non-religious people in adjusted analyses. However, identifying with a religion other than Christianity was associated with slightly less study participation in some models, although sample sizes for these ‘other’ religious are quite low ( Table 1) and the effect sizes were relatively small and often bordering the null.

Religious attendance therefore appears to be ‘missing not at random’ (MNAR, *i.e*., continued participation may depend on religious attendance). As such, when using religious attendance as an exposure or outcome, there is a risk of potential selection bias. Additional sensitivity analyses—such as not at random multiple imputation ( Lee *et al.*, 2021; Tompsett *et al.*, 2018), simulations ( Millard *et al.*, 2021) or Bayesian approaches to model selection ( Du *et al.*, 2022)—may be required to explore the extent of selection bias and whether this may impact the subsequent conclusions drawn ( Griffith *et al.*, 2020; Smith, 2020). Additionally, other aspects of RSBB—belief in God/a divine power and religious affiliation—were frequently associated with continued participation in unadjusted analyses, but these associations were often attenuated to null after adjusting for potential confounders. This suggests that these RSBB factors may be ‘missing at random’ (MAR, *i.e*., adjusting for confounders included here, religious belief and affiliation are not associated with selection). These RSBB factors may not therefore be MNAR, and so are at less risk of causing selection bias; however, to avoid these RSBB variables resulting in selection bias, these demographic and socioeconomic confounders would also need to be controlled for in subsequent analyses. Although some studies have suggested that association with participation would have to be fairly severe for the subsequent bias to cause concern ( Cornish *et al.*, 2021; Pizzi *et al.*, 2011), this needs to be explored on a case-by-case basis. It is hoped that highlighting the potential for selection bias in ALSPAC’s RSBB data will inform future users of the resource.

In addition to these practical implications regarding selection bias, this study also suggests that religious individuals may be more cooperative (using ‘continued participation’ as a measure of cooperation) than non-religious individuals. This supports previous research indicating that religiosity is associated with cooperative behaviour ( Henrich, 2021; Lewis *et al*, 2013). These results do suggest, however, that these cooperative effects may be driven more by religious participation (attending a place of worship more frequently), rather than religious belief or affiliation. If these findings represent a true causal effect, understanding the mechanism(s) by which religious attendance, but not religious belief or affiliation, shapes cooperative behaviour is a key area for future research.

There are several key strengths of this study. First, we use a large, geographically representative population with almost 30 years of longitudinal data, with RSBB measured at baseline and detailed measures of multiple confounders, to explore this topic. Second, we explored these associations in three cohorts of ALSPAC participants (G0 mothers, G0 partners and G1 children), and found similar patterns of results in each. Third, we use a range of RSBB exposures (religious belief, affiliation, and attendance) to explore in detail how various aspects of RSBB impact study participation. Fourth, the use of four different regression models with various sensitivity analyses, all pointing to similar associations, also highlights that these results are robust to multiple model specifications. Together, the strengths of the study design and the consistency of results suggests these results may lend themselves to a causal interpretation; that religious attendance, but not religious belief or affiliation, may cause increased study participation.

Nonetheless, there are several limitations with this study. To begin with, a potential causal interpretation of these results rests on many (largely untestable) assumptions being met. For instance, as with any observational study there is always the potential for residual confounding. This could be due to confounders available in ALSPAC that were missed by our theoretical framework, or potential factors which cause both study participation and RSBB but are either not available in ALSPAC or not available for the majority of participants. For example, personality is only evaluated in later G1 questionnaires and therefore would be missing for many participants. This study also assumes that the confounders included here are in fact confounders ( *i.e.*, causing both the exposures and the outcomes), and not mediators of the RSBB-participation relationship ( *i.e*., caused by RSBB); we have endeavored to select only covariates which are true confounders, but these assumptions may not be correct, in which case the results here may be biased ( Major-Smith *et al.*, 2022).

A related limitation regarding confounders is the use of many proxy measures of variables, such as G0 mother’s IMD and urban/rural status being used for G0 partners. This assumes that the partners live with and/or share wealth with the study mothers, which may be true in many cases but not all. This pertains to another assumption made that the G0 partners did not change over time, this is because the partners were not formally enrolled in ALSPAC but were instead given a questionnaire at the discretion of the study mother. This means that a change in partner could potentially result in different people answering questionnaires and the RSBB data collected at birth potentially being incorrect. While possible, the frequency of this occurring is likely to be low, so any impact of measurement error on the conclusions is likely to be minimal.

The results of this study may also be subject to selection bias, since the questionnaire with questions on RSBB was not the first given to participants and therefore that RSBB factors may have influenced those who answered this questionnaire. However, as only ~10% of G0 mothers did not have baseline RSBB data ( Table 1), any such effects are unlikely to result in a substantial amount of bias. The cohort of G0 partners had slightly more missing RSBB data; however, we do not think this would have a dramatic impact on the results as they are broadly similar to the G0 mother results and across all models. With around 75% of eligible pregnancies enrolled, there a chance of selection bias being caused by original enrollment, however it is believed that the cohort is largely geographically representative ( Boyd *et al.*, 2013).

A further issue concerns potential measurement error. For example, the act of identifying with a certain group may be largely separate from engaging in behaviours typically associated with the group, and the two may have separate impacts on one’s life ( Armitage & Christian, 2003; Dono *et al*., 2010). When applied to religious beliefs, it is possible that many participants may identify as Christian simply because their family are or it is socially desirable ( Abrams, 2001), but do not actually engage in behaviours that more ardent Christians would, *i.e*. attending a place of worship or praying. This is highlighted in this study as 50% of G0 mothers say they believe in a divine power and 80% identify as Christian yet only 14% of mothers attend church a minimum of once a month. This introduces the issue of measurement bias into the study as we cannot be certain asking participants if they believe in God/a divine power or identify as Christian is an accurate measure of religiosity ( Page & Henderson, 2008). This is an issue because many of the theorized mechanisms by which religiosity increases in study participation are centered around engagement in cooperative religious networks and prosocial religious norms ( Norenzayan *et al.*, 2016; Saroglou *et al.*, 2005). As many participants who identify as Christian or believe in a divine power may not engage in religious behaviours, they therefore may not acquire these cooperative norms and consequently act more like those who do not identify as religious in terms of participation in ALSPAC. This could potentially result in the actual associations between study participation and belief in God and/or religious affiliation being attenuated towards the null. Focusing on religious behaviours, and not just religious belief or affiliation, may therefore be especially important when exploring religiosity; as observed here, religious attendance—but not belief or affiliation—was associated with increased participation.

A further key limitation regards the generalizability of these results. First, this study was conducted on a predominantly white and Christian population in the Southwest of the United Kingdom; whether these results are generalizable to other nations, religions and cultures requires further research. Second, given the low numbers of religious non-Christians it was necessary to combine these together into a single ‘other’ category. This may obscure variability in behaviour within this heterogenous group.

A final limitation is that we were unable to assess whether G1 religiosity was associated with study participation. Instead, we had to use maternal RSBB as a proxy for G1 religiosity. While religious beliefs are frequently transmitted from parents to children ( Leonard *et al.*, 2013), transmission is often far from perfect, meaning that measurement error may bias these results. In addition, prior to becoming independent adults, G1 participation in ALSPAC is likely to be determined largely by their mother; the extent to which these measures of G1 participation assess the behaviour of the G1 child, rather than their mother, may be somewhat unclear. However, G1 participation in the most recent questionnaire at age 28—which presumably is largely independent from their mothers—had a similar pattern of results to the other G1 analyses which included participation in childhood. This suggests that the relationships in these other G1 analyses are unlikely to be solely due to the influence of G0 mothers.

In conclusion, we have shown, using repeatedly-collected longitudinal data, multiple models and adjusting for a range of relevant confounders, that religious attendance—but not religious belief or affiliation—is associated with study participation in ALSPAC. This has the potential to result in spurious associations due to selection bias. This work can be used as a basis for future research considering the issue of selection bias due to RSBB in both ALSPAC and wider research on the causes and consequences of religious beliefs and behaviours.

## Data availability

Please see the ALSPAC data management plan which describes the policy regarding data sharing ( http://www.bristol.ac.uk/alspac/researchers/data-access/documents/alspac-data-management-plan.pdf), which is by a system of managed open access. Data used for this submission will be made available on request to the Executive ( alspac-exec@bristol.ac.uk). The datasets presented in this article are linked to ALSPAC project number B3906, please quote this project number during your application.

The steps below highlight how to apply for access to the data included in this study and all other ALSPAC data:

    1. Please read the ALSPAC access policy ( http://www.bristol.ac.uk/media-library/sites/alspac/documents/researchers/data-access/ALSPAC_Access_Policy.pdf) which describes the process of accessing the data and samples in detail, and outlines the costs associated with doing so.

    2. You may also find it useful to browse our fully searchable research proposals database ( https://proposals.epi.bristol.ac.uk/?q=proposalSummaries), which lists all research projects that have been approved since April 2011.

    3. Please submit your research proposal ( https://proposals.epi.bristol.ac.uk/) for consideration by the ALSPAC Executive Committee. You will receive a response within 10 working days to advise you whether your proposal has been approved.

### Extended data

Supplementary information supporting this submission can be found on the Open Science Framework “Religious/spiritual beliefs and behaviours and study participation in a prospective cohort study (ALSPAC) in Southwest England” project page ( https://doi.org/10.17605/OSF.IO/EM36Y).

This project contains the following extended data:

1. “B3906 SuppInfo.pdf” (the supplementary information file)
2. “B3906 STROBE-checklist.pdf” (the completed STROBE cohort study reporting guidelines checklist).
3. “B3906 Stata Script.do” (The Stata script used for analysis).

Data are available under the terms of the Creative Commons Attribution 4.0 International license (CC-BY 4.0).
