# Supplementary material for: The associations between religious/spiritual beliefs and behaviours and study participation in a prospective cohort study (ALSPAC) in Southwest England
Source: Wellcome Open Res. 2024 Jun 27;7:186. Originally published 2022 Jul 12. [Version 2] doi: 10.12688/wellcomeopenres.17975.2 (PMC11234084; doi:10.12688/wellcomeopenres.17975.2)
Supplement: Peer review history

# Reviewer report: Reviewer response for version 2

**DOI:** 10.21956/wellcomeopenres.24916.r88024

**Published:** 2024-07-09

**Authors:** Lluis Oviedo

**Affiliations:** 1 Theological Anthropology, Antonianum University, Rome, Italy

Yes, it is ok for me this new version and the answers from the authors.

Is the work clearly and accurately presented and does it cite the current literature?

Yes

If applicable, is the statistical analysis and its interpretation appropriate?

Yes

Are all the source data underlying the results available to ensure full reproducibility?

Yes

Is the study design appropriate and is the work technically sound?

Yes

Are the conclusions drawn adequately supported by the results?

Yes

Are sufficient details of methods and analysis provided to allow replication by others?

Yes

Reviewer Expertise:

Theology and scientific and empirical study of religion

I confirm that I have read this submission and believe that I have an appropriate level of expertise to confirm that it is of an acceptable scientific standard.

# Reviewer report: Reviewer response for version 2

**DOI:** 10.21956/wellcomeopenres.24916.r88023

**Published:** 2024-07-05

**Authors:** Berihun Dachew

**Affiliations:** 1 School of Population Health, Curtin University, Perth, WA, Australia

Thank you for addressing the comments and suggestions. I have no further comments.

Is the work clearly and accurately presented and does it cite the current literature?

Yes

If applicable, is the statistical analysis and its interpretation appropriate?

Yes

Are all the source data underlying the results available to ensure full reproducibility?

Yes

Is the study design appropriate and is the work technically sound?

Yes

Are the conclusions drawn adequately supported by the results?

Yes

Are sufficient details of methods and analysis provided to allow replication by others?

Partly

Reviewer Expertise:

Epidemiology, Maternal and Child Health, Mental Health.

I confirm that I have read this submission and believe that I have an appropriate level of expertise to confirm that it is of an acceptable scientific standard.

# Reviewer report: Reviewer response for version 1

**DOI:** 10.21956/wellcomeopenres.19925.r80743

**Published:** 2024-05-16

**Authors:** Berihun Dachew, Biruk Tusa

**Affiliations:** 1 School of Population Health, Curtin University, Perth, WA, Australia; 2 School of Population Health, Curtin University, Curtin University (Ringgold ID: 1649), Perth, Western Australia, Australia

Dear Editorial Team,

 Thank you for the opportunity to review the manuscript titled "Religious/Spiritual Beliefs and Behaviours and Study Participation in a Prospective Cohort Study (ALSPAC) in Southwest England." The manuscript is well written and raises an important but often overlooked factor in Epidemiology. Below, we have provided our comments for your review. We hope the comments will help in improving the clarity, rigor, and overall quality of the manuscript.

 Kind regards,

 Berihun Dachew

 Comments:

 Title:

 Suggested title: “The Association between Religious/Spiritual Beliefs and Behaviours and Study Participation in a Prospective Cohort Study (ALSPAC) in Southwest England.”

 Abstract:

- In the methods section, it would be beneficial if the authors mentioned the analytical methods used to assess the association between the outcome and exposure variables.

Introduction:

- The implication of the study is not clear. What would the authors recommend if religious/spiritual beliefs and behaviours are associated with study participation? Are authors encouraging people to follow religious practice, or are they suggesting the next upcoming cohorts to select religious follower participants to reduce loss to follow-up?

Methods:

- Since RSBB data were only collected during pregnancy, but some participants may change their RSBB status over time, leading to misclassification bias. The authors also stated that “The G0 mothers’ RSBB data was used as a proxy for G1 religiosity, as comparable RSBB questions for G1 participants were not asked until adulthood, meaning it would not be possible to assess whether RSBB was associated with continued G1 participation using these data. These variables were chosen to cover a range of religious beliefs and behaviours." This is a significant limitation of the study, increasing the risk of misclassification bias.
- It would be beneficial for the authors to include references to confounding variables drawn from existing literature.
- The authors considered multiple methods of analysis for a single outcome. To reduce complexity, choosing one appropriate best model would enhance the clarity of the article.
- Some important assumptions for the linear model were either not checked or not reported.
- Model adequacy for logistic regression and survival analysis was either not checked or not reported.

**Result and Discussion:**

- In the method section, the authors reported that the linearity assumption failed. Therefore, it seems unnecessary to report results from this model.

**In survival analysis,**

- It is essential to clearly define the outcome of interest, whether it is dropout or continuation. The authors appear to treat both outcomes as exchangeable, which could impact the interpretation of the results.
- Before reporting analytical analyses such as Cox regression, it would be beneficial to first present descriptive statistics, including Kaplan-Meier curve results. This would provide a comprehensive overview of the data and enhance the reader's understanding of the subsequent analytical findings.

Is the work clearly and accurately presented and does it cite the current literature?

Yes

If applicable, is the statistical analysis and its interpretation appropriate?

Yes

Are all the source data underlying the results available to ensure full reproducibility?

Yes

Is the study design appropriate and is the work technically sound?

Yes

Are the conclusions drawn adequately supported by the results?

Yes

Are sufficient details of methods and analysis provided to allow replication by others?

Partly

Reviewer Expertise:

Epidemiology, Maternal and Child Health, Mental Health.

We confirm that we have read this submission and believe that we have an appropriate level of expertise to confirm that it is of an acceptable scientific standard, however we have significant reservations, as outlined above.

# Reviewer report: Reviewer response for version 1

**DOI:** 10.21956/wellcomeopenres.19925.r51514

**Published:** 2022-08-04

**Authors:** Lluis Oviedo

**Affiliations:** 1 Theological Anthropology, Antonianum University, Rome, Italy

I did read with interest this article, as it connects with my own research on religion, prosocial behaviour and coping. In my opinion this is an excellent research that merits to be indexed as soon as possible. The data analysis and the presentation of the framework and conclusions are fine. However, I will suggest some minor nuances.

 The first concerns the wording chosen to describe the main variable under study: "Religious/spiritual beliefs and behaviours". Indeed this is a too broad basket that has been later disentangled into three separate features: beliefs, belonging, and attendance. According to the results, it is apparent that only attendance has a significant effect. This result goes in the same direction to our own research, in which not confession or beliefs, but religious practice becomes the more predictive factor. The recent book of T. Luhrmann "How God Becomes Real" provides an interesting case and explanation for this data.

 The second caveat has to do with the insistence in the article about a possible bias in this sort of longitudinal studies, since, as the data show, more committed religious people tend to answer more the questionnaires along successive waves. This is a curious case: the bias - in my opinion - should not be understood negatively; after all we can always consider and distinguish such a variable and try to measure its weight; we can for instance practice tests to compare those more and those less religious in the same population or sample. I am not sure indeed to be able to see the problem the article raises. 

 I would be grateful if the authors address these two issues in the final version of their article, or in the Discussion section.

Is the work clearly and accurately presented and does it cite the current literature?

Yes

If applicable, is the statistical analysis and its interpretation appropriate?

Yes

Are all the source data underlying the results available to ensure full reproducibility?

Yes

Is the study design appropriate and is the work technically sound?

Yes

Are the conclusions drawn adequately supported by the results?

Yes

Are sufficient details of methods and analysis provided to allow replication by others?

Yes

Reviewer Expertise:

Theology and scientific and empirical study of religion

I confirm that I have read this submission and believe that I have an appropriate level of expertise to confirm that it is of an acceptable scientific standard, however I have significant reservations, as outlined above.
